# Arthroscopic vs. Open-Ankle Arthrodesis on Fusion Rate in Ankle Osteoarthritis Patients: A Systematic Review and Meta-Analysis

**DOI:** 10.3390/jcm12103574

**Published:** 2023-05-20

**Authors:** Alejandro Lorente, Leire Pelaz, Pablo Palacios, Iker J. Bautista, Gonzalo Mariscal, Carlos Barrios, Rafael Lorente

**Affiliations:** 1 Ankle and Foot Surgery Unit, Department of Traumatology and Orthopaedic Surgery, University Hospital Ramón y Cajal, 28034 Madrid, Spain; alejandro.lorentegomez@gmail.com (A.L.); dra_lire@hotmail.com (L.P.); 2 Department of Traumatology and Orthopaedic Surgery, Sanchinarro University Hospital, 28050 Madrid, Spain; pablopalacios@drpalacios.com; 3 Institute of Sport, Nursing, and Allied Health, University of Chichester, Chichister PO19 6PE, UK; i.bautista@chi.ac.uk; 4 Physiotherapy Department, Valencia Catholic University of Valencia, 46900 Valencia, Spain; 5 Institute for Research on Musculoskeletal Disorders, School of Medicine, Valencia Catholic University, 46001 Valencia, Spain; carlos.barrios@ucv.es; 6 Department of Orthopedic Surgery and Traumatology, University Hospital of Badajoz, 06080 Badajoz, Spain; rafaalelorente@hotmail.com

**Keywords:** ankle osteoarthritis, arthroscopic, open surgery, arthrodesis, meta-analysis

## Abstract

Although open surgery is the conventional option for ankle arthritis, there are some reports in the literature regarding the use of the arthroscopy procedure with outstanding results. The primary purpose of this systematic review and meta-analysis was to analyze the effect of the surgery technique (open-ankle arthrodesis vs. arthroscopy) in patients with ankle osteoarthritis. Three electronic databases (PubMed, Web of Science, and Scopus) were searched until 10 April 2023. The Cochrane Collaboration’s risk-of-bias tool was used to assess the risk of bias and grading of the recommendations assessment, development, and evaluation system for each outcome. The between-study variance was estimated using a random-effects model. A total of 13 studies (including *n* = 994 participants) met the inclusion criteria. The meta-analysis results revealed a nom-significant (*p* = 0.072) odds ratio (OR) of 0.54 (0.28–1.07) for the fusion rate. Regarding operation time, a non-significant difference (*p* = 0.573) among both surgical techniques was found (mean differences (MD) = 3.40 min [−11.08 to 17.88]). However, hospital length stay and overall complications revealed significant differences (MD = 2.29 days [0.63 to 3.95], *p* = 0.017 and OR = 0.47 [0.26 to 0.83], *p* = 0.016), respectively. Our findings showed a non-statistically significant fusion rate. On the other hand, operation time was similar among both surgical techniques, without significant differences. Nevertheless, lower hospital stay was found in patients that were operated on with arthroscopy. Finally, for the outcome of overall complications, the ankle arthroscopy technique was a protective factor in comparison with open surgery.

## 1. Introduction

Osteoarthritis (OA) is a common degenerative joint disease that affects the cartilage, bone, and surrounding tissues of joints. It is also known as a degenerative joint disease or wear-and-tear osteoarthritis [1,2]. OA can occur in any joint in the body but commonly affects the knees, hips, spine, and hands [3]. The disease is thought to result from not only the aging process but also from biomechanical and biomechanical change stresses affecting the articular cartilage; however, the exact cause of osteoarthritis is not well known. Indeed, several factors contribute to its development, including age [4], genetics [5], joint injury or overuse [6], and obesity [7,8]. Several studies have identified post-traumatic etiology as the principal cause of ankle arthritis [9]. While the management of OA should be individually guided to satisfy the needs of each patient, the surgical option is reserved for more advanced OA patients and/or for patients where early treatment (i.e., patient education, weight management, and assistive devices) fails [10].

According to the definition of terms in medical subject headings, the surgical fixation of a joint by a procedure designated to accomplish the fusion of the joint surface by promoting the proliferation of the bone cell is called arthrodesis. In this regard, open-surgery arthrodesis represents the traditional option for the treatment of ankle osteoarthritis and related pathologies (i.e., chronic instability and degenerative deformity) due to its effect on pain relief and functional improvements [11]. In recent years, however, ankle replacement has gained more consideration, becoming the preferred treatment for this pathology. A meta-analysis found a greater improvement in function and range of motion when compared to ankle arthrodesis [12]. Nonetheless, the complications following lower-extremity open surgery include infections, wound issues, nerve entrapment, and delayed union and non-union, which could represent an important burden for patient quality of life [13]. On the other hand, the ankle arthroscopic technique represents a valid alternative to open surgery for patients with ankle arthrosis. Although open surgery is the traditional option for ankle osteoarthritis, there are some reports in the literature regarding the use of the arthroscopy procedure with outstanding results, including shorter operative time [14] and hospital stay [15], as well as comparable fusion rates between open vs. arthroscopic interventions [16]. For these reasons, there is still an open debate about the adequacy of which surgical technique (i.e., open vs. arthroscopic) yields better responses in patient outcomes.

The choice of surgical approach, whether arthroscopic or open, may depend on a variety of factors, including the surgeon’s preference, the patient’s condition, and the extent of the surgery required [11]. These inconsistencies and gaps in the literature establish a need for a systematic review that, with the highest scientific rigor, shows the effect of two surgery techniques (i.e., open-ankle arthrodesis vs. arthroscopy) on several clinical outcomes. To date, there are several systematic reviews and meta-analyses where this question was addressed [13,17]. However, some of them fail in study research design classification and others in the statistical analysis approach [18], which could lead to misinterpreting the conclusions obtained. Therefore, the primary purpose of this systematic review and meta-analysis was to analyze the effect of the surgery technique (open-ankle arthrodesis vs. arthroscopy) on fusion rate in patients with ankle osteoarthritis. On the other hand, the second objective of this systematic review and meta-analysis was to analyze the effect of the surgery technique on operation time and length of hospital stay. Finally, our review described the overall complications after the use of both surgical techniques described above.

## 2. Materials and Methods

### 2.1. Study Design

A systematic review and meta-analysis were developed using the Reporting Items for Systematic Reviews and Meta-Analysis (PRISMA) statement guidelines [19]. In addition, the Prisma in Exercise, Rehabilitation, Sport Medicine and Sports Science (PERSiT) was also implemented [20]. The PRISMA checklist is detailed in Supplementary File S1.

### 2.2. Eligibility Criteria

To be included, studies had to adhere to the following criteria: (1) Type of studies: randomized or non-randomized controlled trial where the effect of the surgery technique was assessed. Only studies in English were considered. Conference abstracts were excluded. (2) Type of participant: studies included patients with osteoarthritis, including post-traumatic osteoarthritis, osteoarthritis, and end-stage osteoarthritis, or patients with ankle instability. (3) Types of interventions: open surgery for the intervention group; meanwhile, arthroscopy was the comparison group. (4) Type of outcome measures: the primary outcome of interest was fusion rate. However, in addition to that variable, operation time and length of hospital stay were collected from studies that provided this information. Finally, overall complications were also collected.

### 2.3. Search Strategy

A PICO strategy was used to build search criteria for electronic databases (i.e., PubMed, Web of Science, and Scopus). No restrictions were applied concerning the year of publication. The PICO consisted of terms for open-ankle arthrodesis, arthroscopy, fusion rate, and blood loss. The primary search string used for PubMed was: (“open ankle arthrodesis” [All Fields] OR “open ankle” [All Fields] OR “ankle joint/surgery” [MeSH Major Topic]) AND (“arthroscopy” [All Fields] OR “arthroscopy technique” [All Fields] OR “arthrodesis” [All Fields] OR “minimally invasive” [All Fields]) AND (“fusion rate” [All Fields] OR “Visual analogue scale (VAS)” [All Fields] OR “blood loss” [All Fields] OR “American Orthopaedic Foot and Ankle Society (AOFAS)” [All Fields]). The search strings used for other databases were adapted using the Polyglot Search Translator Tool (https://sr-accelerator.com/#/polyglot, accessed on 4 May 2023) [21] and are reported in Supplementary File S2. The final search date was performed on 10 April 2023. Forward and backward citation tracking of articles that met the eligibility criteria was performed using an online tool (citation chaser) [22].

### 2.4. Methodological Quality and Level of Evidence

Two researchers independently assessed the methodological quality of the studies using a modified version of the Risk of Bias 2 (RoB 2) Cochrane Bias Assessment Tool [23] In the case of disagreement between the scores provided, the primary author made the final decision. RoB 2 was considered in the interpretation of the results by applying the Grading of Recommendations Assessment, Development and Evaluation (GRADE) system. A more extensive description of the risk of bias assessment procedure and the GRADE system is found in Supplementary File S3.

### 2.5. Data Extraction

The following data were extracted: authors, year of publication, research design (i.e., randomized controlled trial (RCT) and non-randomized controlled trial but intervention study (nRCT)), sample size, sex (i.e., male/female), age (i.e., years), body mass index, fusion rate, follow-up period (i.e., months), hospital stay (i.e., days), and overall complications both arthroscopy and open-surgery groups. Data extraction was manually performed by two researchers. Where data were not available or insufficient information was reported, the corresponding author of the studies was contacted by email, with one reminder after 2 weeks if they did not respond to the first email. If the corresponding authors did not reply, the study was discarded.

### 2.6. Statistical Analysis

The sample size and means (or events), standard deviation, 95% confidence intervals (CI_95%_) (if applicable) of fusion rate, complications, hospital stay, and operation time were extracted independently from the included studies. Mean differences (*MD*) were calculated for hospital stay and operation time since all studies were reported in the same units. We first computed a change score within each group and then determined the difference between the change scores between groups using the following equation:

$$MD={Mean}_{arthroscopy}-{Mean}_{open}$$

Finally, the variance of the *MD* was computed as follows [22]:

$$S_{MD}^{2}=\frac{\left(n_{arthr}-1\right)S_{arthr}^{2}+(n_{open}-1)S_{open}^{2}}{n_{arthr}+n_{open}-2}\left(\frac{1}{n_{arthr}}+\frac{1}{n_{open}}\right)$$

where “*S*^2^*_arthr_*” and “*S*^2^*_open_*” denote the variance of the change score for the arthroscopy and open-surgery groups, respectively.

On the other hand, for nominal variables (fusion rate and overall complications), odds ratios (ORs) and 95% of confidence intervals were calculated using the following approach [24]:

$$OR=\frac{a({event}_{arthr})/b({noevent}_{arthr})}{c({event}_{open})/d(no{event}_{open})}$$

The ORs were transformed to *log*-*OR* using the natural logarithm:

$$logOR={log}_{e}(OR)$$

Meanwhile, the standard error of the *log*-*OR* was calculated using the formula:

$${SE}_{logOR}=\sqrt{\frac{1}{a}}+\frac{1}{b}+\frac{1}{c}+\frac{1}{d}$$

The consistency of the effects found was assessed using the I^2^ and τ^2^ tests, with heterogeneity (I^2^) being considered small (<25%), moderate (25–49%), and high (>50%). In addition, Tau-square tests (τ^2^) and prediction interval (PI) were included, because τ^2^ cannot readily point to the clinical implications of the unobserved heterogeneity [25] for ratio variables. The prediction interval allows a better clinical evaluation of the results obtained because it represents the range in which the effect size of a future study conducted on the topic will most likely be. The Egger’s test and a representation of the funnel plot were used to assess small study bias. Variance estimations between studies were calculated using a random-effects model (i.e., Hartung–Knapp/Sidik–Jakman adjustment (HKSJ)) with a 95% confidence interval (CI_95%_). All statistical analyses were performed using statistical software (R version 4.1.9, R Foundation for Statistical Computing, Austria, metaphor and meta-analysis package, general meta-analysis package; risk-of-bias figures were created using Robvis). The standardized mean difference (SMD) was considered trivial (<0.20), small (0.20–0.59), moderate (0.60–1.19), large (1.20–1.99), and very large (>2.00) [26]. RoB 2 figures were created using the Robvis package [26,27].

## 3. Results

### 3.1. Search Results

**Figure 1:**
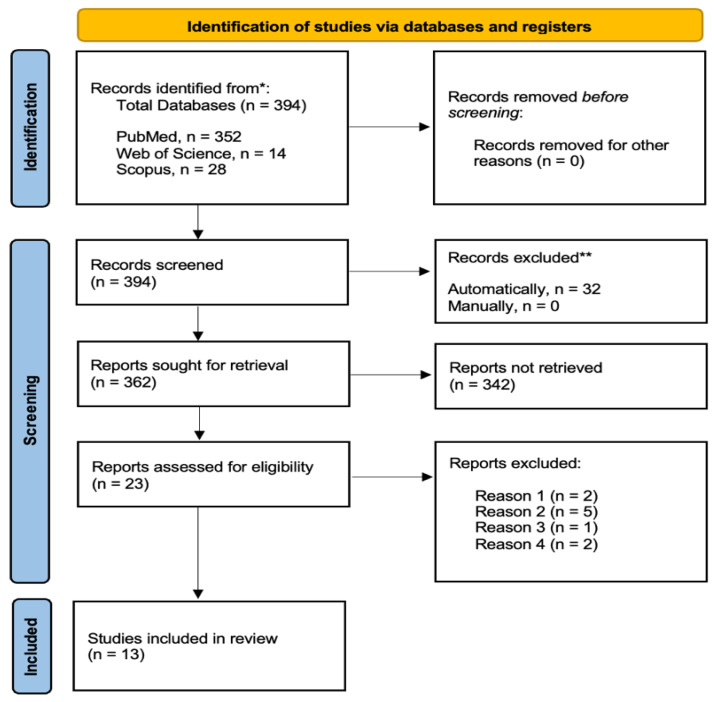
PRISMA flow diagram of the literature search results. * records removed automatically using an online tool (www.sr-accelerator.com, accessed on 4 May 2023). ** records excluded according by reasons.

Figure 1 shows the PRISMA flow diagram with the different phases of the search and selection of studies included in this review. The initial search yielded 394 records. None of the records were removed before screening. After the elimination of duplicates (*n* = 32), another 343 studies were excluded based on abstract and another 10 studies based on full-text assessment (see Supplementary File S4 for more information regarding excluding studies). A total of 13 studies [11,14,15,16,28,29,30,31,32,33,34,35,36] were therefore included in the present review on the effectiveness of open-ankle arthrodesis vs. arthroscopy on our primary outcome (i.e., fusion rate).

### 3.2. Risk-of-Bias Results

**Figure 2:**
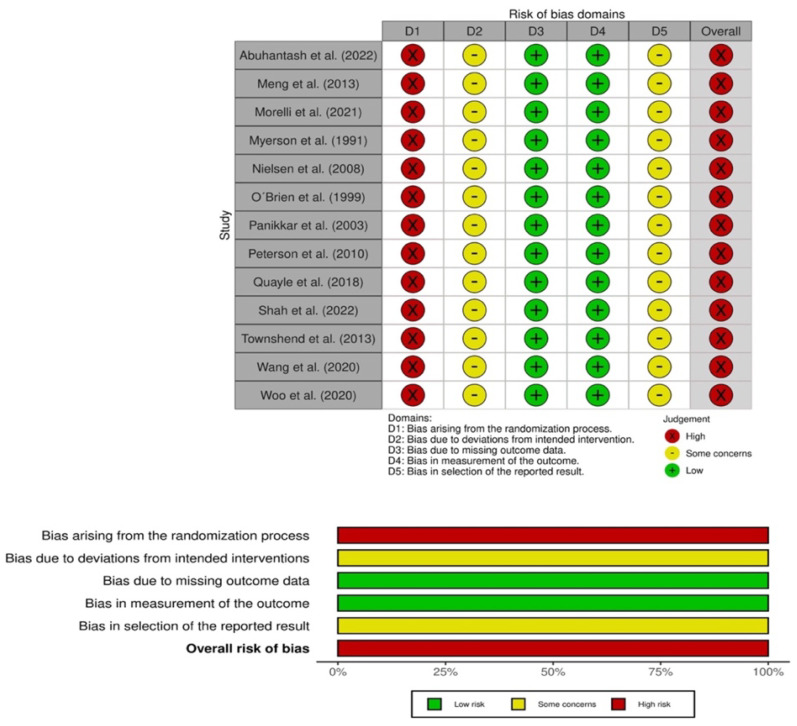
Risk-of-bias assessment for fusion rate traffic light plot and and summary plot [11,14,15,16,28,29,30,31,32,33,34,35,36].

The risk-of-bias scores of included studies are reported in Figure 2 both traffic light and summary plots. A total of 13 studies were analyzed [11,14,15,16,28,29,30,31,32,33,34,35,36]. Figure 2A,B summarizes the risk of bias on fusion rate outcome. From a general point of view, all studies (100%) were at high risk of bias. From the 13 studies analyzed, Domain 1 (randomization procedure) in 13/13 was at high risk of bias, Domain 2 (deviations from the intended intervention) was at high risk of bias in 13/13 (100%), Domain 3 (missing outcome data) was at low risk of bias in 13/13 (100%), Domain 4 (measurement of the outcome) was at low risk of bias in 13/13 (100%), and finally, Domain 5 (selection of the reported results) was 13/13 (100%) at some concerns.

### 3.3. Participants Characteristics

The total sample size across all studies was *n* = 994 participants, where regardless of the operation technique, *n* = 23 (58%) and *n* = 383 (42%) were females and males, respectively. In two studies, the sex was not provided; for that reason, the number of participants was 909. The mean and SD of age were 57.68 ± 6.05 and 57.37 ± 6.49 years for arthroscopy and open surgery, respectively. The body mass index corresponded to 28.07 ± 2.56 and 29.31 ± 4.08 for the arthroscopy and open-surgery groups. A complete description of the patient characteristics is summarized in Table 1.

### 3.4. Fusion Rate

**Figure 3:**
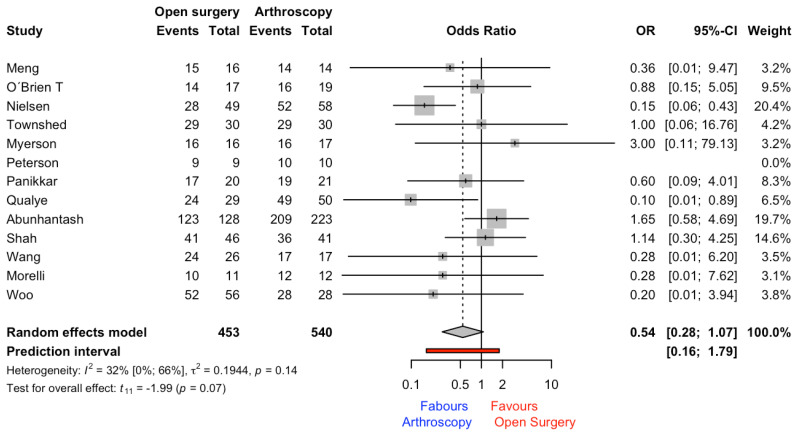
Forest plot for fusion rate outcome [11,14,15,16,28,29,30,31,32,33,34,35,36].

A total of 13 studies yielded a non-significant (*p* = 0.072) rate of fusion of 0.54 (0.28–1.07) for arthrodesis compared with open surgery, including 540 (54%) and 454 (46%) patients for arthroscopy and open surgery, respectively. The OR score corresponded to 0.54 (0.28–1.07). The amount of heterogeneity was cataloged as low (I^2^ = 32%) (see Figure 3).

### 3.5. Operation Time

**Figure 4:**
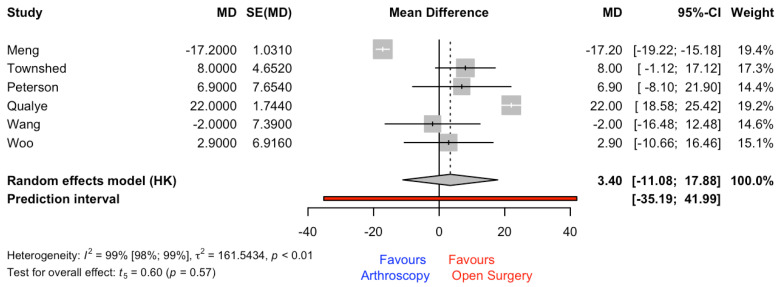
Forest plot for mean difference (MD) (min) for operation time outcome. SE = standard error [14,15,28,33,35,36].

This outcome was reported in a total of eight studies; however, only six studies provided the SD to calculate the MD. The meta-analysis results revealed a non-significant (*p* = 0.573) MD and CI_95%_ of 3.40 min (−11.08 to 17.88) for the open-surgery group. The heterogeneity and prediction interval are shown in Figure 4.

### 3.6. Length of Hospital Stay

**Figure 5:**
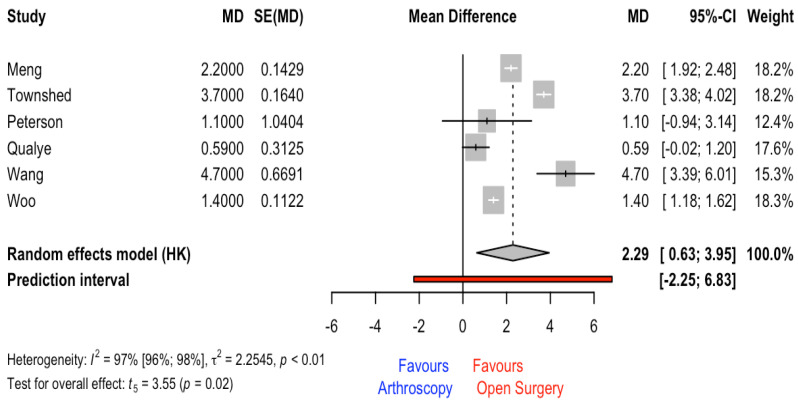
Forest plot for hospital length stay [14,15,28,33,35,36].

This outcome was reported in a total of 10 studies; however, only 6 studies provided the SD to calculate the MD. The meta-analysis results revealed a significant (*p* = 0.017) MD and CI_95%_ of 2.29 days (0.63 to 3.95) for the open-surgery group. The heterogeneity and prediction interval are shown in Figure 5.

### 3.7. Post-Operative Overall Complications

**Figure 6:**
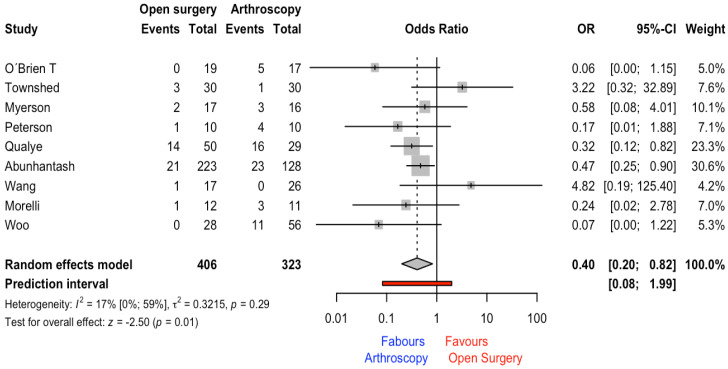
Forest plot for post-operative complications outcome [11,14,16,28,29,30,33,35,36].

A total of 9 studies, including 43 and 66 patients for arthroscopy and open surgery, respectively, reported the number of complications. The meta-analysis results revealed a statistically significant OR and CI_95%_ of 0.40 (0.20 to 0.82) (*p* = 0.012), favoring the arthroscopy surgical technique. The heterogeneity was low (I^2^ = 17%) (see Figure 6).

On the one hand, regarding overall complications, the most common complications described were delayed union, wound infection, non-union, deep infection, tibial entrapment, subtalar osteoarthritis, and tarsal tunnel syndrome in the open-surgery group. On the other hand, non-union, malunion, deep infection, and delayed wound healing were reported for the arthroscopy group.

### 3.8. Heterogeneity Analysis and Publication Bias

**Figure 7:**
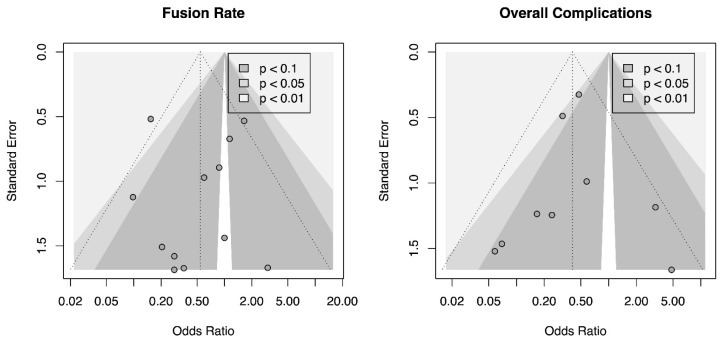
Counter-enhanced funnel plot for fusion rate and overall complications.

Visual analysis of the counter-enhanced funnel plot did not show the presence of publications’ bias fusion rate (A) and overall complications (B) (Figure 7). This was confirmed analyzing Egger’s test for both outcomes (fusion rate intercept = −0.031, CI_95%_ = −1.75 to −1.69, t = −0.036, *p* = 0.972, and overall complications intercept = −0.046, CI_95%_ = −1.30 to −1.21, t = −0.072, *p* = 0.944).

Table 2 summarizes the GRADE evaluation system for the outcomes included in the present systematic review and meta-analysis.

## 4. Discussion

The main aim of this systematic review and meta-analysis was to analyze the effect of the surgery technique (i.e., open surgery vs. arthroscopy) on fusion rate in patients with ankle osteoarthritis. Evidence coming from studies with some concerns or high risk of bias showed that the arthroscopy technique had a non-statistical benefit in comparison to open-ankle surgery. The quality of evidence synthesis was rated as low. On the other hand, when operation time was compared among surgical techniques, the meta-analysis results revealed non-significant differences (3.40 min [11.08 to −17.88]). However, significant differences with an MD of 2.29 days (0.63 to 3.95) favoring the open-surgery group were found. In patients that were operated on with the open-surgery technique, the stance in the hospital was higher in comparison to the arthroscopy group. Finally, regarding overall complications, the meta-analysis results revealed that arthroscopy was a protective factor (OR = 0.47 [0.26 to 0.84]) in comparison with open surgery. In the absence of the homogeneity of studies in the outcomes provided, the preferential use of one of these techniques should be guided by other indicators such as patient characteristics or surgical preferences.

Regarding our primary outcome (i.e., fusion rate), the meta-analysis results revealed that although the arthroscopy surgical technique seems to have acted as a protection factor, no significant differences were found when data were compared with the open-ankle surgery technique. In addition, the GRADE evaluation system cataloged fusion rate and operation time as low and very low overall quality, respectively. This result differs from a recent meta-analysis by Bai et al. [17]. On the one hand, new studies were included in our meta-analysis, such as the study by Abuhantasth et al. [11], where a total of 351 patients were treated (*n* = 223 for arthroscopy and *n* = 128 for open surgery). On the other hand, there are several studies that were included in the study of Bai et al. [17], where it was not possible to find the references (impossible to access Chinese electronic databases), which represents a problem of replicability.

Arthroscopy, in comparison with open surgery, requires only small incisions, which means there is less soft-tissue damage and scarring. This can lead to less pain and a faster recovery time. Our results showed that the use of arthroscopy was more beneficial than open surgery (OR = 0.54), but non-significant differences were found in fusion rate (see Figure 3) and in operation time (see Figure 4). It is important to highlight that these results came from studies with high risk of bias in the first domain (i.e., bias arising from the randomization process). It should be considered that, in all studies included in the present systematic review and meta-analysis, the patient division across groups was made according to a specific criterion (i.e., surgeon preferences or other factors). For example, in the study conducted by Woo et al. [36], the decision of which surgical procedure was performed was based on surgeon preference, as well as the study by Abuhantasth et al. [11], which revealed that the surgeon decided on which operation technique to employ on the basis of the anatomy, deformity, and patient comorbidities. These facts could affect the fusion rate and the operation time. The surgeon’s expertise could have an effect on operation time and on fusion rate outcomes.

The total number of complications across groups was 43 and 66 for the arthroscopy and open-surgery groups, respectively. However, when this result was adjusted by the total number of patients, the mean and SD of overall complications were 12% ± 0.08 and 27% ± 0.18 (OR = 0.47, see Figure 6). However, deep infection was reported in both surgical techniques; in this sense, Shah et al. [34] concluded that for patients with a remote history of infection, open-ankle arthrodesis may be preferable.

Based on the primary findings of this study, when the fusion rate outcome was analyzed, there was a beneficial use of the arthroscopy surgical technique in comparison with open surgery. However, it is important to highlight that non-significant differences were found between these surgical techniques. Overall, the use of arthroscopy in ankle arthrodesis can provide several advantages over traditional open surgery, resulting in a faster, less painful recovery with fewer complications. However, as with any surgical procedure, the choice of approach should be made in consultation with the patient’s surgeon, taking into account individual factors such as the patient’s medical history, level of physical activity, and overall health. While arthroscopic ankle arthrodesis offers several advantages over open surgery, there are also some potential disadvantages to consider, for instance, limited visualization; technical difficulty; limited accessibility; risk of complications, such as infection, nerve damage, and blood vessel injury; and limited weight-bearing capacity. However, arthroscopic ankle arthrodesis has demonstrated its advantages, and it is important to highlight that ankle joint replacement is currently the gold standard for ankle osteoarthritis. Ankle arthrodesis is technically less demanding, but patients have limited function. Whereas joint replacement showed better function and range of motion compared with ankle arthrodesis, patient satisfaction showed no difference [12].

There are some limitations in the present systematic review that need to be carefully addressed before interpreting the results obtained. Firstly, there was a large heterogeneity of outcomes across the included studies; meanwhile, some studies included functional scales and pain assessment while other studies did not. The lack of agreement regarding the outcomes assessed creates the necessity for a clinical guideline to be systematic in the outcomes reported. On the other hand, the risk-of-bias analysis of the included studies in this meta-analysis revealed the necessity for studies with a randomization process and an assessor blinded to the patient groups. Finally, arthroscopic ankle arthrodesis is often recommended for well-aligned cases, whereas open fusion is indicated to treat malaligned arthritic ankles. This fact may introduce bias in the interpretation of the results, as the lower complication rate may be attributed to less complex cases and not to the surgical approach itself. In addition, it should be mentioned that the studies do not report on the implants used for fixation, which may also have influenced the results. These major findings could affect the primary outcomes.

## 5. Conclusions

While both arthroscopic and open surgery can be effective for ankle arthrodesis, the evidence found suggests that arthroscopic surgery may produce similar or even better outcomes with several potential advantages over open surgery (i.e., fewer overall complications). In conclusion, our findings show that studies with some concerns or high risk of bias provided a better but non-statistically significant fusion rate in patients that underwent arthroscopic arthrodesis in comparison with open surgery. The quality of evidence was rated as low. On the other hand, operation time was not different among surgical techniques, although a lower hospital stay was found in patients that were operated on with arthroscopy. Finally, for the outcome of overall complications, the ankle arthroscopy technique was a protective factor in comparison with open surgery. For these reasons, the choice of surgical approach should be based on the careful consideration of the individual patient’s condition and the surgeon’s experience and preference.

## Figures and Tables

**Table 1:** Baseline characteristics of the included studies.

Study	Total, N	Arth N	Open N	Age Art	Age Open	Sex Arth (M/F)	Sex Open (M/F)	BMI Arthr	BMI Open	Follow-Up (Months) Arthr	Follow-Up (Months) Open
Meng et al., (2013) [28]	30	14	16	NR	NR	0/16	0/16	NR	NR	NR	12
O’Brien et al., (1999) [16]	36	19	17	47.3	44.6	9/10	7/10	NR	NR	NR	NR
Nielsen et al., (2008) [31]	107	58	49	51	53	31/27	34/15	NR	NR	12	12
Townshend et al., (2013) [35]	60	30	30	59.4	54.7	20/10	11/19	27.4	29.6	24	24
Myerson et al., (1990) [30]	33	17	16	NR	NR	10/7	9/7	NR	NR	NR	NR
Peterson et al., (2010) [33]	20	10	10	56.2	54.8	6/4	5/5	32.11	37.36	NR	NR
Panikkar et al., (2003) [32]	41	21	20	68	65	12/9	17/3	NR	NR	9	6
Quayle et al., (2018) [15]	79	50	29	57	61.9	37/13	19/10	28.9	28	12	12
Abunhantaash et al., (2022) [11]	351	223	128	57.9	57.1	150/73	81/47	29.1	28.8	39	48
Shah et al., (2022) [34]	87	41	46	NR	NR	NR	NR	NR	NR	4	5
Wang et al., (2020) [14]	43	17	26	54.76	55.35	10/7	16/10	26.55	28.93	32	35
Morelli et al., (2021) [29]	23	12	11	64.6	67	5/7	8/3	23.8	23.6	NR	NR
Woo et al., (2019) [36]	84	28	56	60.6	60.2	9/19	18/38	28.64	28.9	NR	NR

Note: NR = not reported.

**Table 2:** Grading of Recommendations Assessment, Development and Evaluation system (GRADE) on fusion rate, operation time, hospital stay, and overall complications.

Outcome	Summary of Findings				Quality of Evidence Synthesis (GRADE)			
	k	*n*	OR (CI9_5%_)	Direction Effect Compared to Control	Imprecision	Inconsistency	Risk of Bias	Overall Quality
Fusion Rate	13	995	0.54 (0.28 to 1.07)	↔	−1	None	−1	●●○○○ Low
Operation Time	6	316	3.40 (−11.08 to 17.88)	↔	−1	−1	−1	●○○○○ Very Low
Hospital Stay	6	316	2.29 (0.63 to 3.95)	↓	−1	−1	−1	●●○○○ Low
Overall Complications	9	413	0.47 (0.26 to 0.84)	↓	None	None	−1	●●●○○ Moderate

Note: CI confidence interval, GRADE Grading of Recommendations Assessment, Development and Evaluation; k, number of studies; *n*, number of participants; SMD, standardized mean difference.

## Data Availability

All data generated or analyzed during this study are included in this published article (and its Supplementary Materials).

## Supplementary Materials

The following are available online at https://www.mdpi.com/article/10.3390/jcm12103574/s1, File S1: Prisma Checklist. File S2: Search strings. File S3: Risk of Bias and Grading of Recommendation Assessment, Development and Evaluation system. File S4: Excluded studies with reasons. References [19,37,38,39,40,41,42,43,44,45,46] are cited in Supplementary Materials.
